# Burden, causes, and risk factors of perinatal mortality in Eastern Africa: a protocol for systematic review and meta-analysis

**DOI:** 10.12688/gatesopenres.13915.1

**Published:** 2022-09-23

**Authors:** Yohanis Alemeshet Asefa, Lars Åke Persson, Anna C. Seale, Nega Assefa

**Affiliations:** 1 London School of Hygiene & Tropical Medicine, London, UK; 2 Haramaya University, Harar, Ethiopia; 3 Health System and Reproductive Health Research Directorate, Ethiopian Public Health Institute, Addis Ababa, Ethiopia; 4 Warwick Medical School, University of Warwick, Coventry, UK

**Keywords:** Keywords: Perinatal mortality, stillbirths, early neonatal mortality, East Africa

## Abstract

**Background:** Although global mortality rates in children under 5 years have decreased substantially in the last 30 years, there remain around 2.6 million stillbirths and 2.9 million neonatal deaths each year. The majority of these deaths occur in Africa and South Asia. To reduce perinatal deaths in East Africa, knowledge of the burden, but also the risk factors and causes of perinatal deaths are crucial. To the best of our knowledge, reviews have previously focused on the burden of perinatal deaths; here we aim to synthesize evidence on the burden, causes, and risk factors for perinatal mortality in East Africa.

**Methods:** We will conduct a systematic literature search in Medline, Web of Science, EMBASE, Global Health, SCOPUS, Cochrane Library, CINAHL, HINARI, African Index Medicus, African Journals Online (AJOL), and WHO African Regional Office (AFRO) Library. We will include studies from 2010 to 2022, and to facilitate the inclusion of up-to-date data, we will request recent data from ongoing surveillance in the region, such as the Child Health and Mortality Prevention Surveillance (CHAMPS) network and Health and Demographic Surveillance sites (HDSS sites). To assess the quality of included studies we will use the Joanna Briggs Institute quality assessment tool for observational and trial studies. We will analyze the data using STATA version 17 statistical software and assess heterogeneity and publication bias by Higgins’ I ^2^ and funnel plot, respectively.

**Conclusions:** This systematic review protocol will search for published studies, and seek unpublished data, on the burden, causes, and risk factors of perinatal mortality in East Africa. Findings will be reported and gaps in the evidence base identified, with recommendations, with the ultimate aim of reducing perinatal deaths.

**Protocol registration:** PROSPERO-CRD42021291719.

## Introduction

Despite the decline of global mortality rates in children under 5 years from 93 per 1000 live births in 1990 to 38 per 1000 live births in 2019, there are currently 2.6 million stillbirths and 2.9 million neonatal deaths each year ^1– 3^. The vast majority of these deaths occur in low- and middle-income countries, in Africa and South Asia ^4^.

The Every Newborn Action Plan (ENAP) was launched in 2014, which targets the reduction of the neonatal mortality rate (NMR) to 12 or fewer per 1,000 live births and stillbirths to 12 or fewer per 1,000 births in all countries by 2030 ^5^. However, Africa has the highest stillbirth rate, and the slowest improvement worldwide ^6^. Thus, given current changes, it would take over 160 years for a pregnant woman in Africa to have the same chance of having an alive baby as a woman in high-income nations now ^7^. Further, Africa has the slowest reduction rate of neonatal mortality and the highest neonatal mortality rate in the world, at 27 (25–32) deaths per 1,000 live births, followed by South Asia at 23 (21–26) deaths per 1,000 live births ^8– 10^.

More than three-quarters of all newborn deaths are from preventable and treatable conditions. The most common causes are prematurity, intrapartum-related deaths (including birth asphyxia) and neonatal infections ^11, 12^. Counting the number of deaths precisely and consistently classifying causes and risk factors for perinatal mortality is one of the objectives of the Every Newborn Action Plan, which is essential to inform effective interventions ^5^. In addition, using consistent definitions and classification systems is important to interpret the causes of perinatal deaths ^13, 14^. Hence, understanding the burden, causes and risk factors of perinatal mortality is critical to reduce perinatal deaths in East Africa towards the 2030 targets.

To the best of our knowledge, previous reviews have focused on the burden of perinatal deaths, and none addressed the causes of perinatal mortality ^15, 16^. Moreover, these reviews did not attempt to incorporate unpublished studies. Therefore, this systematic review and meta-analysis aims to synthesize the most recent evidence (published and unpublished reports) on the burden, causes, and risk factors of perinatal mortality in East Africa published from 2010 to 2022.

### Research questions

This systematic review and meta-analysis will answer the following questions:

1.   What is the overall perinatal mortality rate and how does this vary in different contexts (geographic location, study setting) in East Africa?

2.   What are the causes of perinatal mortality in East Africa?

3.   What are the risk factors for perinatal mortality in East Africa?

## Methods

### Protocols used for reporting and protocol registration

The design and implementation of this systematic review will adhere to the Preferred Reporting Items for Systematic Review and Meta-Analysis Protocols (PRISMA-P) 2015 statement ^17^ and reporting of findings will follow the Preferred Reporting Items for Systematic review and Meta-Analyses (PRISMA-2020) updated guideline. ^18^ The protocol for this review was registered on PROSPERO ( CRD42021291719).

### Eligibility criteria

Studies for this systematic review and meta-analysis will be selected based on the criteria specified below.

### Inclusion and exclusion criteria

We will include both published and unpublished studies, that report perinatal mortality (stillbirth and/or early neonatal death), its causes and/or risk factors of perinatal mortality. No restrictions will be imposed on language of publication, sex, or ethnicity of participants. This study will include studies that have been conducted in East Africa and published between January, 2010 – June, 2022.

We will exclude studies which are reviews, or published outside of the study area and time period (before 2010 and after June, 2022). We will also exclude studies that focus on specific populations (e.g., high-risk mothers). Studies will be excluded if extracting data is not feasible after appropriate attempts to seek the full text and contact the corresponding author where needed. We will exclude studies that are limited in methodology (inappropriate statistical analysis or methods used to control confounders).

### PECO search guide

**Population:** All births (both livebirths and stillbirths) with ≥500g/≥22 weeks of gestation and newborn deaths within the first week after birth (0–6 days) ^19^.

**Exposure:** Determinants or risk factors of perinatal mortality. The determinants or risk factors are characteristics or exposures that increase the likelihood of perinatal mortality. These may be related to distal, underlying, or proximal determinants.

**Comparison:** The reported reference group for each determinant or risk factor in each study (e.g., perinatal mortality in mothers with antenatal care versus mothers with no antenatal care).

**Outcome:** Perinatal mortality rate, which is defined as “the total number of deaths of a fetus with birth weight of 500 grams or more or a gestational age of 22 completed weeks of age or more until the 7 ^th^ day after delivery per 1000 live birth” ^20^. The other outcomes for this study are the causes and risk factors of perinatal mortality.

### Study designs

All observational studies (cross-sectional, case-control, prospective cohort and retrospective studies) and community-based trials which reported the magnitude of perinatal mortality and/or its cause or risk factors will be included.

### Study setting and time frame

Studies that have been conducted in East Africa, which encompasses the following countries; Burundi, Comoros, Djibouti, Eritrea, Ethiopia, Kenya, Madagascar, Malawi, Mauritius, Mozambique, Rwanda, Seychelles, Somalia, Somaliland, South Sudan, Sudan, Tanzania, Uganda, Zambia, and Zimbabwe according to United Nations ^21^ will be considered. Both community-based and facility-based studies will be used for this study. This study will be conducted from October 2022 to February 2023. 

### Years and language considered for study recruitment

All studies published from January 1, 2010 to June 30, 2022 and all articles reported in any language will be considered for this study. We will consult a specific language expert for translation for studies in a language the authors do not speak.

### Publication status

All studies that fulfill the eligibility criteria will be considered regardless of their publication status (published, and unpublished or grey literature). To access unpublished reports of likely high relevance and quality, up-to-date data will be requested from large surveillance studies, forming an investigator group.

### Information sources

The databases searched to identify published research articles will be Medline, Web of Science, EMBASE, Global Health, SCOPUS, Cochrane Library, CINAHL, HINARI, African Index Medicus, African Journals Online (AJOL), and WHO African Regional Office (AFRO) Library. In addition to this, a manual search will be performed to retrieve unpublished studies and grey literature via Google Scholar, Google and institutional repositories of higher education institutions, which are found in East Africa and outside the region that have joint projects in East Africa. An investigator group from large studies with ongoing surveillance in the region will be requested from Child Health and Mortality Prevention Surveillance (CHAMPS) and Health and Demographic Surveillance sites (HDSS) to facilitate the inclusion of the most up-to-date data. Three CHAMPS networks in the region, namely Harar and Kersa, Ethiopia, Siaya and Kisumu, Kenya, and Manhiça, Mozambique ^1^, and HDSS from Ethiopia (Harar and Kersa, Dabat, Butajira, Arba Minch and Gilgel Gibe HDSS), Kenya (Nairobi, Kilifi, Mbita, Kombewa and Kisumu HDSS), Malawi (Blantyre, Karonga HDSS), Mozambique (Chokwe and Manhica HDSS), Tanzania (Magu, Rufiji, Bagamoyo, Ifakara, Korogwe, Moshi and Pemba HDSS), Uganda (Awach; Gulu, Iganga/Mayuge, Kyamulibwa, Rakai and Toro HDSS), Zambia (Lusaka HDSS) ^22, 23^ will be asked to join the investigator group if they have appropriate data, which they are able to contribute. 

### Search strategy

We will search the electronic databases above, based on the following concepts: perinatal mortality (stillbirth and/or early neonatal mortality), causes of perinatal mortality, stillbirths or early neonatal mortality, risk factors for perinatal mortality, stillbirths or early neonatal mortality, study design (cross-sectional, case-control, cohort and community-based trial) and location and geographic setting (countries of Eastern Africa), and published covering the time period from January 1, 2010 to June 30, 2022.

The search will be conducted in appropriate search fields of electronic databases, and with sensitive searches that combine text words with indexing terms. Both free-text words (including spelling variants, synonyms, related terms, plurals, acronyms, truncations, wildcards, and proximity operators) and appropriate subject headings will be used. We will use Boolean operators ‘AND’ and ‘OR’ to connect and focus a search by combining subject headings and keywords.

Various combinations of the following key terms will be used to identify papers on the burden of perinatal mortality, its cause and determinants in Eastern Africa: ‘perinatal mortality’, ‘perinatal death(s)’, stillbirth(s), stillborn(s), ‘fetal death(s)’, ‘fetal demise’, ‘fetal mortality’, ‘neonatal death(s)’, ‘infant mortality’, and ‘East Africa’ ( Table 1). We will also identify studies that were cited by others (descendent search strategy).

**Table 1.** Summary of search strategy in Ovid Medline electronic database.

Component	Search terms
1	Perinatal Mortality/ or stillbirth/ or fetal death/ or infant mortality/
2	((perinat* or f?etal or f?etus* or infant* or neonat*) adj5 (death* or mortalit* or demise)).mp.
3	(stillbirt* or stillborn* or adverse birth outcome* or pregnancy outcome* or perinatal outcome*).mp.
**4**	**1 Or 2 OR 3**
**5**	exp Africa, Eastern/
6	(eastern Africa or Burundi or Comoros or Djibouti or Kenya or Madagascar or Malawi or Mauritius or Mozambique or Rwanda or Seychelles or Somalia or Somaliland or South Sudan or Tanzania or Uganda or Zambia or Zimbabwe).mp.
**7**	**5 OR 6**
**8**	**4 and 7**
**9**	**limit 8 to yr=”2010 - 2022”**

## Study records

### Data management

Articles will be searched using different electronic databases and imported to EndNote software version X20 using each of the databases’ citation manager to facilitate review process and exclusion of duplicated studies.

### Selection of studies

After importing studies to Endnote X20, duplicates will be excluded. Titles and abstracts of remaining studies will be screened by Y.A.A., then abstracts of selected studies will be exported to Covidence review management software for full-text screening ^24^. Full-text articles will be independently screened by two investigators (Y.A.A. and N.A), and when there is uncertainty, a third reviewer (L.A.P or A.C.S) will make a final decision. The total number of studies identified, screened, eligible and included in the study will be described, and the reason for exclusion at each stage of the study selection process will be explained. A single failed eligibility criterion is sufficient for a study to be excluded from a review. Results from comparable groups of studies will be combined into a statistical meta-analysis using STATA-17 software ^25^.

### Data collection process

Required information for the systematic review will be extracted and summarized using the Joanna Briggs Institute and Cochrane data extraction template. Information on the title, author, publication year, study design, study setting (rural vs. urban), study type (community-based vs. hospital-based), sample size, study participants, study period, sampling methods, and outcome of interest (definition of outcomes) will be extracted. When we extract data regarding perinatal mortality rate, we will note the denominator used by studies (live births or total births) and we will also collect the stillbirth and early neonatal death rates separately. Furthermore, we will extract the time of death for stillbirths; antepartum or intrapartum (fresh or macerated) if reported. In addition to this, we will look at the ascertainment of causes and risk factors in each study and the classification system will be recorded where available. The measures of association (odds ratio or relative risk with their respective confidence intervals) for each risk factor will be extracted and included in meta-analyses where feasible.

### Data items

**Perinatal mortality rate**: is the sum of stillbirths and deaths in the first week of life (0–6 days) per 1000 total birth (both live and stillbirths) ^19^.

**Stillbirth rate:** is fetal death at ≥500g/≥22 weeks gestation or ≥1000g /≥28 weeks gestation by WHO for general statistics and international comparison respectively per 1000 total births ^26^.

**Early neonatal mortality**: deaths among live births during the first week (0–6 days) of life ^27^.

**Causes of perinatal mortality:** are any condition/s with a reasonable mechanism likely to lead to the death of the fetus or early neonate and it is classified as the underlying cause, immediate cause, and main maternal cause) ^28, 29^.

**ICD-PM:** is the WHO application of ICD-10 to deaths during the perinatal period, which provides a standardized system for classifying perinatal mortality based on time of death as antepartum (before the onset of labor), intrapartum (during labor but before delivery) or neonatal (the first week after delivery), and it also links the contributing maternal condition, if any, with perinatal death ^28^.

**Figure 1.**
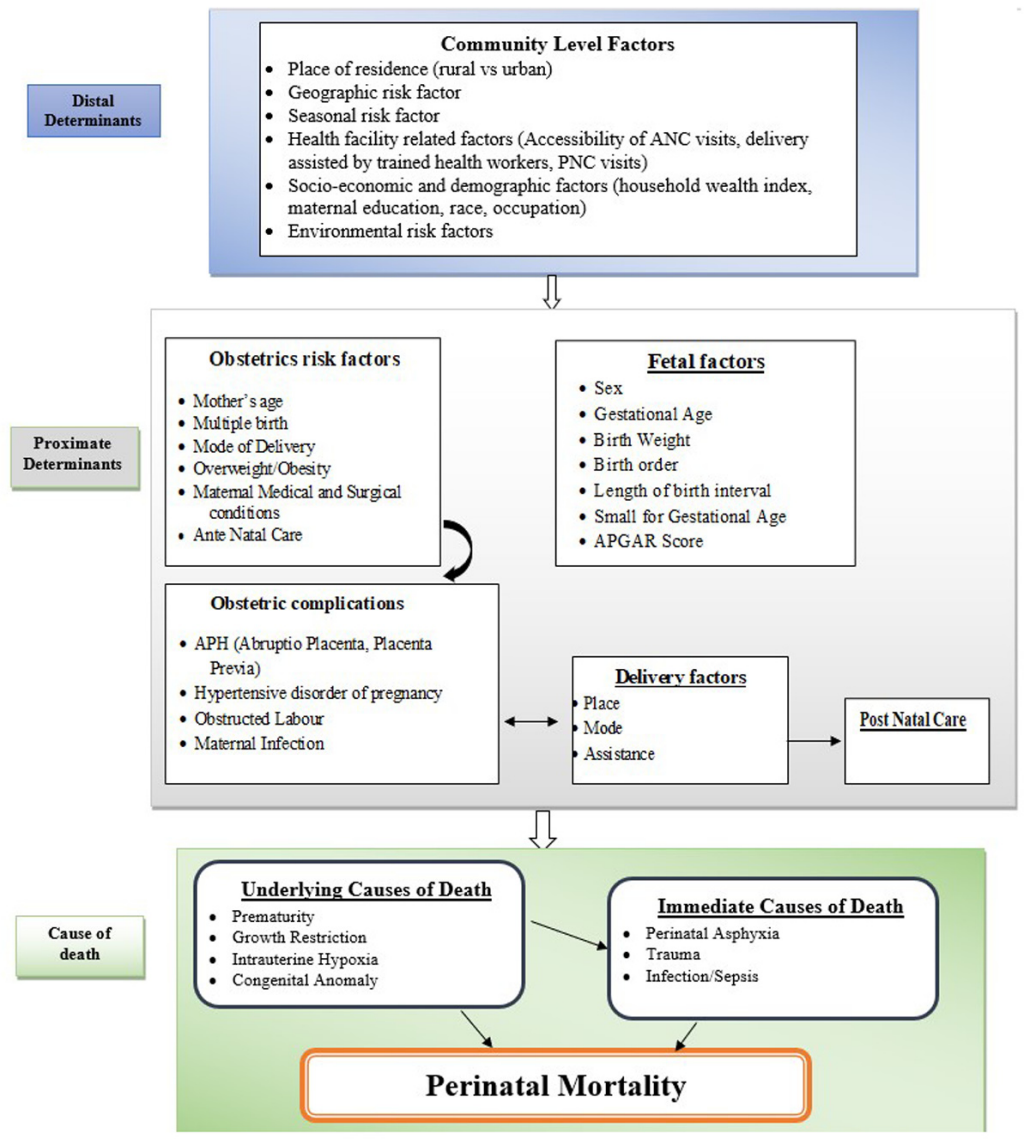
Conceptual framework for causes and risk factors of perinatal mortality, adapted and modified from Mosley and Chen ^33^.

**Determinants or risk factors**: are characteristics associated with, but not obviously causal for, stillbirths or early neonatal deaths, such as advanced maternal age ^30^. ( Figure 1)

### Outcomes and prioritization

The perinatal mortality rate will be the primary outcome measure; it is calculated by dividing the number of fetal deaths after 22 weeks of gestation or weighing more than 500g and neonatal deaths in the first week after delivery by the total number of births (stillbirths and live births) that have been included in the study (sample size) ^5, 19^. The second outcome is the determinants (risk factors) that are associated with perinatal mortality among the study subjects; factors associated with perinatal mortality will be socio-demographic and economic factors, maternal factors, fetal factors, health service-related factors, and environmental factors. The third outcome measure of the study will be the causes of perinatal mortality, which can be classified as the underlying cause (perinatal asphyxia or hypoxia, infection or sepsis, preterm birth complications, congenital anomalies), immediate cause (perinatal asphyxia or hypoxia, infection or sepsis, preterm birth complication, and birth trauma), and main maternal factors (complications of placenta, cord or membranes and maternal medical or surgical conditions, mainly associated with hypertensive disorder of pregnancy) of death ^1, 31^ ( Figure 1). Furthermore, if any other classification systems were utilized in the included studies, we will also attempt to synthesize them.

### Critical appraisal of individual studies

We will assess the methodological quality of included studies using the Joanna Briggs Institute quality assessment tool for observational and trial studies ^32^. The Joanna Briggs Institute critical appraisal tool for cross-sectional, case-control and cohort studies comprises 8, 10 and 11 questions, respectively. The tool supports an assessment of sample representativeness of the target population, participant recruitment, adequacy of the sample size, detailed description of the study subjects and study setting, appropriate method of the statistical analysis, objective criteria in the measurement of the outcome variable and identification of subpopulation, reliability, and identification of confounding variables ^34^.

Each item for each study will be judged as Yes (1) and No (0). When the information provided is not adequate to make a judgment for a specific item, we will grade that item with a ‘No’ (0). Each study will be graded depending on the number of items judged ‘Yes’ (1) as low-risk bias (≥ 7), medium-risk bias (5 to 6), or high-risk bias (≤ 4) for cross-sectional studies, low-risk (≥8), medium-risk (5 to 7) and high-risk (< 5) for case-control studies, and low-risk (≥ 9), medium-risk (6 to 8) or high-risk (≤ 5) for cohort studies, and trials will be treated as cohort studies. 

### Data synthesis

The study selection processes will be summarized using a PRISMA flow diagram, and for studies which are excluded the reason will be described and explained ^17^. A narrative synthesis will be used to summarize all studies included in the study and characteristics like study population, cause of perinatal mortality, and identified risk factors will be summarized in a descriptive table.

### Meta-analysis

If appropriate perinatal mortality rates from different studies with a common definition of perinatal mortality will be pooled together to provide a single summary (pooled perinatal mortality rate) estimate using STATA-17 software. Further, we will calculate the pooled risk ratio for the risk factors of perinatal mortality using the random effect model as it assumes that the observed variation of effect size is because of real differences ^35^. The syntax “metaforestplot” will be used to generate forest plots with their corresponding weights, as well as the pooled rate across studies and its corresponding 95% Confidence Intervals (Cl).

### Heterogeneity test

To examine the magnitude of the variation between studies statistical heterogeneity test will be assessed by Higgins’ I ^2^. The I ^2^ test measures level of statistical heterogeneity between studies; the values of <25 %, 25–50 %, 50–75 % and >75% are to mean very low, low, medium and high heterogeneity respectively ^36^. Since heterogeneity is expected in this study because of the differences in perinatal mortality rate across different settings, random effect model will be used. If heterogeneity is significant (I ^2^>50%), sub-group analysis, meta-regression or meta-analysis will be conducted to investigate sources of heterogeneity and if meta-analysis is not possible, a narrative synthesis will be conducted.

### Subgroup and sensitivity analysis

Sub-group analysis will be conducted based on study design, study type (community-based or facility-based), publication status (published or unpublished), study setting (rural vs. urban), geographic stratification, publication year, and study quality score (low or high score).

Sensitivity analysis will be performed to assess the robustness of a pooled estimate. We will use the single study omission analysis to test the robustness of a pooled estimate, and a study will be considered to have no influence on the pooled prevalence if the pooled estimate without it lies within the 95% confidence limits of the overall pooled prevalence ^37, 38^. Furthermore, although most countries use the definition of perinatal mortality for international comparison, which is fetal deaths after 28 weeks of gestation or weighing more than 1000g till 7 ^th^ day after birth, in the primary analysis we will try to capture studies that were done using the WHO definition for general statistics (≥500g or ≥22 weeks of gestation). Then, the analysis will be repeated using the perinatal mortality definition for international comparison.

### Publication bias

We will inspect funnel plots subjectively and Egger’s test objectively to assess publication bias. subjectively and evidence of publishing bias will be suggested by an asymmetrical funnel plot. Evidence of publishing bias will be suggested by an asymmetrical funnel plot and a p-value < 0.1 ^39, 40^.

## Conclusion

This review will provide evidence on the burden, causes, and risk factors of perinatal mortality in East Africa. Published data and unpublished reports will be included, and estimates of the burden, causes and risk factors will be compared according to geographic location, study type, and study setting. The review will provide more information on this key topic, identify gaps and make recommendations, with the aim of informing interventions to prevent perinatal deaths. The findings of the study will be shared with the participating surveillance sites, and disseminated through national and international conferences, and peer-reviewed publication.

## Study status

We have developed search strategies and data extraction tools, and the collection and screening of published articles began in August 2022. Data collection from Investigator groups for unpublished reports and overall analysis will be completed by January and February 2023, respectively.

## Data Availability

No data are associated with this article. figshare: PRISMA-P checklist for ‘Burden, causes, and risk factors of perinatal mortality in Eastern Africa; a protocol for systematic review and meta-analysis’, https://doi.org/10.6084/m9.figshare.20627859 ^41^ Data are available under the terms of the Creative Commons Zero "No rights reserved" data waiver (CC BY 4.0 Public domain dedication).
