# Supplementary material for: Burden, causes, and risk factors of perinatal mortality in Eastern Africa: a protocol for systematic review and meta-analysis
Source: Gates Open Res. 2024 May 8;6:123. Originally published 2022 Sep 23. [Version 2] doi: 10.12688/gatesopenres.13915.2 (PMC11258368; doi:10.12688/gatesopenres.13915.2)
Supplement: Peer review history

# Reviewer report: Reviewer response for version 2

**DOI:** 10.21956/gatesopenres.17085.r37376

**Published:** 2024-07-18

**Authors:** Kim J. C Verschueren

**Affiliations:** 1 Utrecht University, Utrecht, The Netherlands

Thank you for the opportunity to review this protocol for an important systematic review. I strongly encourage that this review is conducted, with the recommendations of some minor revisions. It is great that the authors will report articles in all languages (as there may be several in French, Arabic or Swahili).

 1. Objective should be to synthesize evidence on the prevalence, timing (antepartum, intrapartum, unknown), causes, risk factors of perinatal deaths in East-Africa. "The burden" is too general (and difficult to assess as this goes beyond the deaths). Consider to also include 'lessons learned' or 'recommendations' described in literature.

 2. In background the authors mention ENAP goal of 12/1000 by 2030; the action plan itself mentions 10/1000 for each by 2035, please review.

 3. Consider including neonatal deaths until 28 days as some studies define perinatal mortality including late neonatal deaths as well.

 4. Why did the authors decide to include studies until 2022 and not until 2024? This should be updated. Please clarify why 2010 was chosen as the starting point.

 5. Causes are classified with different classification systems; it is not clear how the authors will assess the causes and reclassify them. Why did the authors choose the classification system from Mosley and Chen (2003) and not the WHO-validated ICD-PM classification? (while they do mention it under data items?). I would suggest them, considering that they have to reclassify anyway, to use the latter.

 6. Consider describing not only the relative risk or odds ratio, but also the absolute risk, as this can be clinically more relative.

 7. Perinatal death rate has denominator 'total births' (not live births). 

 8. Please include mode of delivery in your review, as every cesarean performed on a baby who dies, should be considered a CS too much (considering the risks a previous CS scar carries in future pregnancies), as well as the under-utilisation of ventouse (which may save some babies as it is quicker, and reduce many CS).

 9. I recommend the authors not to look too much into 'risk factors / medical determinants' of perinatal deaths, as they are well known and vary not very much between contexts. Many important variables will also likely not be available, which can be absolutely contributing (for example maternal malnutrition, severe anaemia, malaria during pregnancy, etc). It is important to find out and explain why the high perinatal death rate is high in this context: referral system (primary to secondary), referral criteria for high-risk, maternity waiting home, quality of ANC (not just number), quality of 'skilled birth attendant', number of birth attendants per number of births per day, availability of medication/tests during ANC, presence of medical specialist / hospital in case of complications, access and infrastructure, number of women with previous CS and % of VBACs (at higher risk in subseq pregnancy), the manner of fetal monitoring, the possibility to perform ventouse, etc etc) and to develop clear recommendations which interventions are likely the most helpful to reduce perinatal deaths - and these are not similar to the risks (we cannot prevent births from primiparas, nor multiple gestation, but we can improve the management). It is for this reason that we recommend and strongly encourage the authors to go beyond the numbers to really make impact !

 All the best to the authors.

Is the study design appropriate for the research question?

Yes

Is the rationale for, and objectives of, the study clearly described?

Yes

Are sufficient details of the methods provided to allow replication by others?

Partly

Are the datasets clearly presented in a useable and accessible format?

Not applicable

Reviewer Expertise:

Maternal mortality and near miss, and perinatal mortality in low- and middle-income settings. Cesarean sections in LMIC.

I confirm that I have read this submission and believe that I have an appropriate level of expertise to confirm that it is of an acceptable scientific standard, however I have significant reservations, as outlined above.

# Reviewer report: Reviewer response for version 2

**DOI:** 10.21956/gatesopenres.17085.r36767

**Published:** 2024-06-19

**Authors:** Anna Akselsson

**Affiliations:** 1 Sophiahemmet University, Stockholm, Sweden

The revisions are appropriate.

Is the study design appropriate for the research question?

Yes

Is the rationale for, and objectives of, the study clearly described?

Yes

Are sufficient details of the methods provided to allow replication by others?

Partly

Are the datasets clearly presented in a useable and accessible format?

Not applicable

Reviewer Expertise:

Stillbirth, fetal movements, antenatal care

I confirm that I have read this submission and believe that I have an appropriate level of expertise to confirm that it is of an acceptable scientific standard.

# Reviewer report: Reviewer response for version 1

**DOI:** 10.21956/gatesopenres.15208.r34567

**Published:** 2023-08-25

**Authors:** Anna Akselsson

**Affiliations:** 1 Sophiahemmet University, Stockholm, Sweden

Thank you for giving me the opportunity to review this study protocol. It is important that this type of study is focusing on a low resource setting in where most of the worlds perinatal mortality occur. The authors aim to address the burden, causes, and risk factors of perinatal mortality in East Africa and are planning to include both unpublished and published data. The protocol is well written and address an important topic. Thank you for conducting this study.

 I have some concerns that follow below.

**Introduction:** Well written and includes the most important information. However, research question nr 1 is contradictory to one of the exclusion criteria. The sentence “We will also exclude studies that focus on specific populations (e.g., high-risk mothers).” in exclusion criteria versus “What is the overall perinatal mortality rate and how does this vary in different contexts (geographic location, study setting) in East Africa?”.

**Question:** Can you be more specific in exclusion criteria of when to exclude specific populations?

**Methods:** Clearly written and structured well. I miss information about how you obtain unpublished data and how you will handle missing values in the dataset, are you planning to handle missing values by imputation? I guess a lot of studies from East Africa will have many missing values to consider. A lot of potential risk factors and confounders may not be available in the studies you include. Will you publish a statistical analysis plan later on describing this matter?

**Question:** How will you obtain unpublished data and how are you planning on handle missing data?

 Figure 1 is good and clarifies the process, “Ante Natal Care” should be “Antenatal care”

**Question:** In figure 1, can you check the spelling and change where necessary?

Is the study design appropriate for the research question?

Yes

Is the rationale for, and objectives of, the study clearly described?

Yes

Are sufficient details of the methods provided to allow replication by others?

Partly

Are the datasets clearly presented in a useable and accessible format?

Not applicable

Reviewer Expertise:

Stillbirth, fetal movements, antenatal care

I confirm that I have read this submission and believe that I have an appropriate level of expertise to confirm that it is of an acceptable scientific standard, however I have significant reservations, as outlined above.

# Response

**Published:** 2024-04-14

**Authors:** Yohanis Alemeshet

**Affiliations:** London School of Hygiene & Tropical Medicine, Ethiopia

**Thanks very much for reviewing our paper. Our responses to the specific points are discussed in the following bullet points.**

**Introduction: Well written and includes the most important information. However, research question nr 1 is contradictory to one of the exclusion criteria. The sentence "We will also exclude studies that focus on specific populations (e.g., high-risk mothers)." in exclusion criteria versus "What is the overall perinatal mortality rate and how does this vary in different contexts (geographic location, study setting) in East Africa?".**

**Question: Can you be more specific in exclusion criteria of when to exclude specific populations?**

- Thank you for your comment and question. We plan to exclude studies that were conducted on specific populations, for instance, high-risk mothers, that don’t reflect the population, and frequently lack a population denominator. However, if studies report the population denominator (vs the facility denominator) or report the effect size after comparing with low-risk mothers, we will not exclude them.

**Methods: Clearly written and structured well. I miss information about how you obtain unpublished data and how you will handle missing values in the dataset, are you planning to handle missing values by imputation? I guess a lot of studies from East Africa will have many missing values to consider. A lot of potential risk factors and confounders may not be available in the studies you include. Will you publish a statistical analysis plan later on describing this matter?**

**Question: How will you obtain unpublished data and how are you planning on handle missing data?**

- We will try to get unpublished internal reports and/or aggregate data from HDSS sites to complement published sources, using our connections to investigators in the region.
- We will assess the missing data and then consider the strategies for handling it, including consideration of imputation, if appropriate. We have incorporated this into the meta-analysis section. *“For unpublished data from the internal reports/ aggregate data from HDSS sites, We will assess the gaps in the data and deliberate on effective strategies to manage them. This may encompass the possibility of imputation, if appropriate.”*

**Figure 1 is good and clarifies the process, "Ante Natal Care" should be "Antenatal care"**

**Question: In figure 1, can you check the spelling and change where necessary?**

 Thank you for this. Now, “Ante Natal Care” has been corrected into “Antenatal care.

# Reviewer report: Reviewer response for version 1

**DOI:** 10.21956/gatesopenres.15208.r33334

**Published:** 2023-07-20

**Authors:** Vivek V Shukla

**Affiliations:** 1 The University of Alabama at Birmingham, Birmingham, Alabama, USA

The current study aims "to synthesize evidence on the burden, causes, and risk factors for perinatal mortality in East Africa." The study's authors are commended for their work; I have a few suggestions for consideration to improve the manuscript and the overall research.

 Title: The title of the meta-analysis is clear and concise, providing a good overview of the study's focus.

 Abstract: The abstract summarizes the study objectives, methods, and expected outcomes. However, it lacks specific details regarding the population, interventions, comparisons, and outcomes (PECO) framework, which are essential for understanding the scope of the study.

 Introduction: The introduction could benefit from providing more information on perinatal mortality in East Africa. For instance, it would be helpful to include statistics or prevalence rates of perinatal mortality in the region to emphasize the significance of the problem. Additionally, discussing the socioeconomic and healthcare factors specific to East Africa that may contribute to perinatal mortality rates would provide context and enhance the reader's understanding.

 Significance and implications: The introduction could provide a statement on the importance of the study and the potential impact on research and practice. It would be beneficial to highlight how the meta-analysis results can contribute to improving perinatal health outcomes in East Africa, inform policy decisions, or guide future research directions.

 Rationale for the study: The introduction lacks a clear and concise statement of the research gap or rationale for conducting the meta-analysis. It is important to explicitly state why a meta-analysis is needed for the specific region "East Africa" and how it will contribute to the existing body of knowledge.

 Methods:

 Search Strategy: The comprehensive search strategy includes multiple databases and sources to ensure broad coverage of relevant studies. Specific search terms and Boolean operators are helpful in enhancing transparency and reproducibility.

 Eligibility Criteria: The inclusion and exclusion criteria are well-defined, specifying the types of studies, population, interventions, comparisons, and outcomes of interest. Eligibility criteria are clearly described, including the inclusion of both published and unpublished studies. However, it would be helpful to provide a justification for the selected time frame (January 2010 to June 2022) and discuss potential limitations or biases that might arise from excluding studies published before 2010 or after June 2022.

 Language Consideration: It is mentioned that articles reported in any language will be considered, but it would be helpful to mention whether language translation services will be used and the process of extraction for such studies.

 Data Collection: The data extraction process is adequately described, mentioning the key information to be collected. However, the title and abstract screen by a single investigator is sub-optimal. Adding a second reviewer to screen and a third to resolve conflicts would be a better approach.

 Definitions:  Stillbirths defined as "fetal death at ≥500g/≥22 weeks gestation or ≥1000g /≥28 weeks gestation" is confusing. How do the authors plan to ensure comparability across studies?

 The critical appraisal plan is robust.

 Risk of Bias Assessment: The protocol mentions using the Joanna Briggs Institute quality assessment tool for observational and trial studies. Please clarify how the risk of bias will be evaluated and incorporated into the analysis. Will there be any secondary analyses with studies that have been identified as having a low risk of bias? Consider conducting a sensitivity analysis to explore the impact of different inclusion criteria or the exclusion of specific studies on the overall findings.

 Results: A descriptive paragraph elaborating the expected results would be helpful for readers.

 Discussion: I suggest presenting the findings of the published meta-analysis on the subject. What factors did they identify, their impact, and how would the proposed analyses add to the existing literature and its likely impact? I would also add a section on limitations and how the authors have tried to address the limitations.

 Overall, this proposal is of significant importance, featuring commendable writing, and tackling a crucial research topic.

Is the study design appropriate for the research question?

Yes

Is the rationale for, and objectives of, the study clearly described?

Yes

Are sufficient details of the methods provided to allow replication by others?

Yes

Are the datasets clearly presented in a useable and accessible format?

Not applicable

Reviewer Expertise:

Perinatal Epidemiology

I confirm that I have read this submission and believe that I have an appropriate level of expertise to confirm that it is of an acceptable scientific standard, however I have significant reservations, as outlined above.

# Response

**Published:** 2024-04-14

**Authors:** Yohanis Alemeshet

**Affiliations:** London School of Hygiene & Tropical Medicine, Ethiopia

**Thank you very much for reviewing our paper. Our responses to the specific points are discussed in the bullet points following the raised comments/questions.**

**Abstract:** **The abstract summarises the study objectives, methods, and expected outcomes. However, it lacks specific details regarding the population, interventions, comparisons, and outcomes (PECO) framework, which are essential for understanding the scope of the study.**

- Thank you for this comment. Specific details of the population, exposure comparisons and outcomes are now included in the abstract. *"The study population includes all fetuses and newborns from ≥22 weeks of gestation (birth weight ≥500gm) to 7 days after birth, with reported causes or/and determinants as exposure and perinatal mortality (stillbirths and/or early neonatal deaths) as an outcome. *

**Introduction** **: The introduction could benefit from providing more information on perinatal mortality in East Africa. For instance, it would be helpful to include statistics or prevalence rates of perinatal mortality in the region to emphasise the significance of the problem. Additionally, discussing the socioeconomic and healthcare factors specific to East Africa that may contribute to perinatal mortality rates would provide context and enhance the reader's understanding.**

- In the second paragraph, we have incorporated more information specific to East Africa, including some statistics and relevant factors like socioeconomic and healthcare factors specific to the region. 

**Significance and implications: The introduction could provide a statement on the importance of the study and the potential impact on research and practice. It would be beneficial to highlight how the meta-analysis results can contribute to improving perinatal health outcomes in East Africa, inform policy decisions, or guide future research directions.**

- At the end of the introduction, the following statements are added to indicate the significance and implication of this study: ***"*** *Hence, in this systematic review, we aimed to describe the burden, causes and risk factors of perinatal mortality in East Africa, using the most up-to-date information, to determine progress in achieving the ENAP 2030 targets and direct policymakers to allocate resources to the likely most effective interventions to prevent preventable perinatal deaths.*"

**Rationale for the study: The introduction lacks a clear and concise statement of the research gap or rationale for conducting the meta-analysis. It is important to explicitly state why a meta-analysis is needed for the specific region "East Africa" and how it will contribute to the existing body of knowledge.**

- The rationale for conducting this study in East Africa is now more clearly indicated in the second paragraph by adding the following statement – *". . . Perinatal mortality in the region was one of the highest, with 34.5 deaths per 1000 births* *^1^* *. East Africa also has the weakest economy across Sub-Saharan Africa, and utilisation of reproductive health services (facility deliveries, skilled delivery assistance, and 4+ antenatal visits) in East and Central Africa is lower than in other areas of Sub-Saharan Africa* *^2,3^* *. Reducing perinatal mortality in this region is critical; counting the number of deaths precisely and consistently classifying causes and risk factors for perinatal mortality (one of the objectives of the Every Newborn Action Plan), is essential to inform effective interventions* *^4^* *."*

**Methods:** **Search Strategy: The comprehensive search strategy includes multiple databases and sources to ensure broad coverage of relevant studies. Specific search terms and Boolean operators are helpful in enhancing transparency and reproducibility.**

- Thank you for this comment. The specific search terms and Boolean operators used in Medline were included in the protocol, and to enhance transparency and reproducibility, we will also report the search strategies with specific terms and Boolean operators used in each database when we submit the systematic review results. We have also stated this in the protocol under the search strategy section.

**Eligibility Criteria: The inclusion and exclusion criteria are well-defined, specifying the types of studies, population, interventions, comparisons, and outcomes of interest. Eligibility criteria are clearly described, including the inclusion of both published and unpublished studies. However, it would be helpful to provide a justification for the selected time frame (January 2010 to June 2022) and discuss potential limitations or biases that might arise from excluding studies published before 2010 or after June 2022.**

- We decided to consider the timeframe from 2010 until the end of 2022, because the aim of this study is to review the up-to-date information in perinatal mortality, and to ensure that we use sufficient data. Therefore, the following statement is added under the sub-title Years and language considered for study recruitment *". . . because it aims to examine up-to-date information regarding perinatal mortality and the advancements made towards attaining the 2030 goals, whilst ensuring sufficient years are assessed to maximize data included in the analysis.“ *

**Language Consideration: It is mentioned that articles reported in any language will be considered, but it would be helpful to mention whether language translation services will be used and the process of extraction for such studies.**

- We plan to consult a specific language expert to translate other languages that authors do not speak, and we will also use language translation softwares.

**Data Collection: The data extraction process is adequately described, mentioning the key information to be collected. However, the title and abstract screen by a single investigator is sub-optimal. Adding a second reviewer to screen and a third to resolve conflicts would be a better approach.**

- Thank you for this, We have corrected this as " *Titles and abstracts of remaining studies will be screened by Y.A.A., and N.A*"

**Definitions:  Stillbirths defined as "fetal death at ≥500g/≥22 weeks gestation or ≥1000g /≥28 weeks gestation" is confusing. How do the authors plan to ensure comparability across studies?**

- We are considering this broad definition to look at the variation of definitions across different studies; however, in our meta-analysis, we will only consider studies with similar definitions (≥1000g /≥28 weeks gestation).

**The critical appraisal plan is robust.**

**Risk of Bias Assessment: The protocol mentions using the Joanna Briggs Institute quality assessment tool for observational and trial studies. Please clarify how the risk of bias will be evaluated and incorporated into the analysis. Will there be any secondary analyses with studies that have been identified as having a low risk of bias? Consider conducting a sensitivity analysis to explore the impact of different inclusion criteria or the exclusion of specific studies on the overall findings.**

- Thank you for this, in order to check the risk of bias we will use the Risk Of Bias In non-randomized Studies – of Exposure (ROBINS-E), and we will consider a sensitivity analysis to investigate how variations in the inclusion and exclusion of high-risk bias studies can affect the overall. A statement describing this is added under the critical appraisal of individual studies section.

**Results: A descriptive paragraph elaborating the expected results would be helpful for readers.**

**Discussion: I suggest presenting the findings of the published meta-analysis on the subject. What factors did they identify, their impact, and how would the proposed analyses add to the existing literature and its likely impact? I would also add a section on limitations and how the authors have tried to address the limitations.**

- We tried to consider both the expected outcome and discussion under the Discussion and Conclusion section added the following paragraph *"Evidence shows that East Africa has the weakest economy across Sub-Saharan Africa, and utilisation of reproductive health services (facility deliveries, skilled delivery assistance, and 4+ antenatal visits) in East and Central Africa is lower than in other areas of Sub-Saharan Africa.*
- *Although there is no specific systematic review and meta-analysis in Eastern Africa, a meta-analysis of demographic and health surveys in Sub-Saharan Africa indicated that the pooled perinatal mortality in East Africa was 34.5 (95% CI: 32.2, 36.8) per 1000 births. However, the data from demographic and health surveys suffered from underreporting as data is collected retrospectively (deaths happening in the past five years). Another study conducted in Sub-Saharan Africa reported that perinatal mortality in East Africa was 49.88 (28.60, 71.18) per 1000 births and low birth weight, primiparity, history of perinatal loss, multiple gestation, preterm birth, and birth interval <2 years were identified as determinants of perinatal mortality 4. Although it is crucial to consider studies that report only stillbirths or early neonatal deaths, this study did not consider specific studies on either stillbirths or early neonatal deaths. Therefore, this review will consider all studies in perinatal mortality, including stillbirths and early neonatal death-only studies, and the analysis will be done accordingly. Further, this study will shed light on the causes of perinatal mortality, including whether studies use a recently introduced unified classification system (ICD-PM)."*
